# An Interesting Case of Coronavirus NL63 Encephalitis Diagnosed in a 14-Year-Old Immunocompetent Female: A Case Report and Literature Review

**DOI:** 10.7759/cureus.62229

**Published:** 2024-06-12

**Authors:** Ahsan A Saeed, Hadi Helali, Moza Alhammadi

**Affiliations:** 1 Medicine, Mohammed Bin Rashid University of Medicine and Health Sciences, Dubai, ARE; 2 Pediatrics, Al Jalila Children's Specialty Hospital, Dubai, ARE; 3 Infectious Diseases, Al Jalila Children's Specialty Hospital, Dubai, ARE

**Keywords:** literature review of disease, coronavirus, viral encephalitis, encephalitis, hcov-nl63

## Abstract

Human coronavirus NL63 (HCoV-NL63) belongs to the human coronavirus family but is distinct from other common coronaviruses such as HCoV-043, HCoV-229E, and SARS-CoV-1 and SARS-CoV-2 viruses. It causes a mild upper respiratory tract infection, affecting children and adults. The usual symptoms associated with the HCoV-NL63 infection are vomiting, a runny nose, and a sore throat. In vivo, HCoV-NL63 showed neurotropism as it can be detected in the CSF, through which it disseminates into the brain and the spinal column. Herein, we describe the case of a 14-year-old female patient who initially presented with disorientation and a drop in consciousness level and was admitted as a case of encephalitis to the pediatric intensive care unit.

## Introduction

Human coronavirus NL63 (HCoV-NL63) is a mild respiratory tract infection belonging to the human coronavirus family, which consists of seven distinct viruses such as HCoV-229E, HCoV-OC43, HCoV-HKU1, HCoV-NL63, SARS-CoV-1 and SARS-CoV-2, and MERS [1]. While human coronaviruses have been first identified as early as the 1960s [2], HCoV-NL63 is a relatively recent discovery, first identified in 2004 in the Netherlands [3]. A report by the Norwegian Institute of Public Health indicates the mean prevalence for HCoV-NL63 at 1% (seven out of 13 regions) to about 5% (West Africa) of all respiratory infections; it showed no difference in the rate of upper vs. lower respiratory tract infection, indicating the whole respiratory tract is predisposed to infection [4]. These viruses are capable of affecting adults and children, causing a wide variety of respiratory, enteric, hepatic, and neurological conditions that can be severe, especially in the elderly and immunocompromised [5].

Initially, human coronaviruses were believed to cause only mild upper respiratory tract infections, which, in some cases, would expand to severe respiratory tract infections [5]. It is now known that all seven of the human coronaviruses, including HCoV-NL63, have associations with the CNS. In infants and children, the most common symptoms are febrile seizures, with adults manifesting non-focal neurological and constitutional symptoms. HCoV-NL63 uses the ACE2 receptor. Therefore, the neurotropism and neurovirulence of the virus are dependent on the CNS cell’s expression of the receptor [6].

The literature shows that while mononuclear circular cells can be infected, meningoencephalitis is rare [6]. This is evident as, while various epidemiological pediatric studies have been conducted regarding HCoV-NL63, mostly minimal neurological presentations were reported, and of those that were mentioned, none of them were recorded to have been diagnosed with meningitis [7,8]. Herein, we describe the case of a 14-year-old female patient who initially presented with disorientation and a drop in consciousness level. She was admitted as a case of encephalitis to the pediatric intensive care unit, and upon nasopharyngeal swab testing, she was positive for HCoV-NL63.

## Case presentation

The patient was a 14-year-old immunocompetent girl in her usual state of health until three days before her admission, when she developed a fever, cough, and nasal congestion after returning from a holiday abroad 10 days earlier. On the day of admission, she started complaining of headaches and experienced multiple episodes of non-projectile, non-bloody vomiting. She was started on supportive treatment but subsequently began moaning, developed cycling movements of her limbs, became unaware of her surroundings, and then lost consciousness. She was electively intubated in the emergency department due to concerns about her ability to maintain her airway independently. Following this, she was transferred to the pediatric intensive care unit.

A CT scan was performed and reported as normal. A full septic workup, including a lumbar puncture, revealed an elevated WBC count in the CSF of 194 × 10^6^/L and an elevated CSF protein level of 58 mg/dL (Table 1). All cultures were negative except for a nasopharyngeal swab PCR, which was positive for HCoV-NL63. The child was initially started on IV ceftriaxone, vancomycin, and acyclovir. Acyclovir was discontinued almost immediately after HSV1 and HSV2 PCRs were negative, and the antibiotics were discontinued 48 hours after the final cultures returned negative (Table 2).

**Table 1:** CSF studies with reference ranges

Component	Patient value	Reference range
CSF protein (mg/dL)	58	15-40
CSF WBC (L)	194 × 10^6^	0-7 × 10^6^

**Table 2:** Results of the meningitis/encephalitis panel via multiplex PCR PCR, polymerase chain reaction

Pathogen	Result
Cytomegalovirus	Not detected (negative)
Enterovirus	Not detected (negative)
Herpes simplex virus 1	Not detected (negative)
Herpes simplex virus 2	Not detected (negative)
Human herpesvirus 6	Not detected (negative)
Human parechovirus	Not detected (negative)
Varicella zoster virus	Not detected (negative)
*Escherichia coli *K1	Not detected (negative)
Haemophilus influenzae	Not detected (negative)
Listeria monocytogenes	Not detected (negative)
Neisseria meningitidis	Not detected (negative)
Streptococcus agalactiae	Not detected (negative)
Streptococcus pneumoniae	Not detected (negative)
Cryptococcus neoformans/gattii	Not detected (negative)

**Figure 1:**
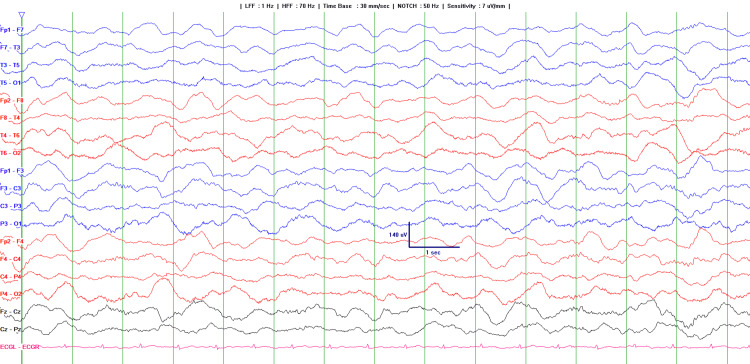
The EEG shows diffuse, generalized slowing in the delta range with no focal or diagnostic epileptiform abnormalities, suggesting either a sedation effect or diffuse encephalopathy.

**Figure 2:**
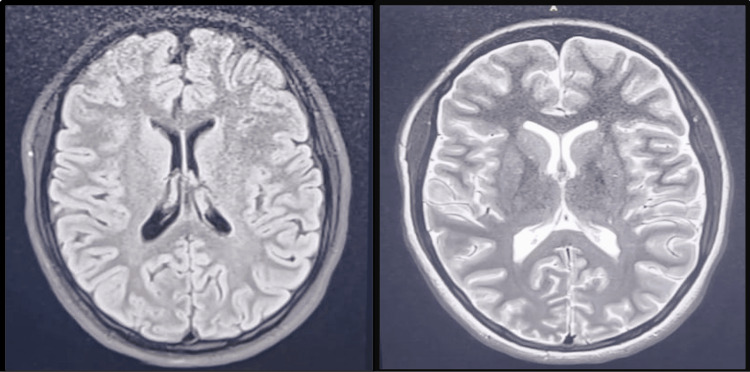
Axial T2 WI through the basal ganglia level appears unremarkable Axial T2 WI: axial T2-weighted images

EEG done on the second day of admission showed diffuse, generalized slowing in the delta wave range with no focal or diagnostic epileptiform abnormalities (Figure 1). The brain MRI performed on the third day of admission was normal, except for an incidental finding of a tiny, uncomplicated colloid cyst at the foramen of Monro (Figure 2). The child was gradually weaned off sedation and was extubated to room air on the fifth day of admission. She regained full consciousness, recognized her parents, and returned to her usual baseline. She was discharged home after a 24-hour observation. Unfortunately, the patient was lost to follow-up.

## Discussion

Despite the surge of research about coronaviruses in the past few years, there is still a large literature gap in the clinical manifestations of HCoV-NL63 in the CNS, especially in pediatrics. Previous studies have suggested that HCoV-NL63 has a temporal inclination for particular seasons [7], but a study by Abdul-Rasool and Fielding concluded that HCoV-NL63 has no seasonal predilection [9]. HCoV-NL63 occurs at a rate of between 1.2% and 2.6% in children [7,8] and in 1% of adult cases (in most regions) [4].

Murine models of HCoV-OC43 demonstrate that human coronaviruses are neurovirulent, as they cause productive neuronal infection with apoptosis and a persistent neurologic deficit. In vitro models show axonal neuron-to-neuron propagation of the virus [6]. This model can be applied to HCoV-NL63 due to its similar pathogenesis to HCoV-OC43 and can demonstrate how HCoV-NL63 can disseminate into the CNS system, leading to neurological symptoms such as febrile seizures, disseminated encephalomyelitis, and meningoencephalitis [10].

After a thorough review of the literature, and to the best of the author’s knowledge, only one other case of encephalopathy was associated with the HCoV-NL63 infection. Yuan and Chunjie describe a 40-year-old man with HCoV-NL63 with cognitive impairment as the first symptom [11]. A reverse transcription polymerase chain reaction was positive for HCoV-NL63 in the CSF, and a positive sputum sample also confirmed the infection. The CT scan showed corpus callosum and paraventricular low-density lesions, and the EEG showed diffuse slow waves with bilateral epileptic discharges in the brain. He was treated with IV immunoglobulin and developed severe complications requiring antiseizure medication and a tracheostomy. After a month of treatment, the patient was discharged but was stated to have poor memory and was unresponsive.

In stark contrast, in this case of the 14-year-old immunocompetent female, the CSF was negative for pathogens, and HCoV-NL63 was only confirmed through a positive nasopharyngeal swab. The patient had a normal CT scan and an EEG with no epileptiform signals. Her condition resolved on its own with only supportive treatment, as previously described.

A scoping review by Singer et al. on long-term neurodevelopmental surveillance concludes that there is a significant gap in the knowledge of the long-term effects of coronaviruses on the neurodevelopment of afflicted children [12].

## Conclusions

HCoV-NL63 is a common respiratory tract infection with neurotropism and neurovirulence, especially in children and even immunocompetent patients, such as in the presented case. While coronavirus encephalopathy is rare, clinical recognition can be delayed due to a lack of prominent cellular inflammation in neuronal necrosis and misidentification of the disease’s etiology. Therefore, clinicians should not rule out neurological comorbidities when dealing with respiratory tract infections at any time of the year. Modeling the virulence of HCoV-NL63 on that of HCoV-OC43, it can be proposed that perhaps axonal neuron-to-neuron transfer may be a reason as to why, in this case of encephalitis, the CSF sample tested negative for HCoV-NL63, whereas the nasopharyngeal swab was positive. Long-term patient follow-up is advised due to the unknown effects of coronavirus encephalopathies on long-term neurodevelopment in children.
